# The Use of Omics in Untangling the Effect of Lifestyle Factors in Pregnancy and Gestational Diabetes: A Systematic Review

**DOI:** 10.1002/dmrr.70026

**Published:** 2025-01-12

**Authors:** Kai Liu, Georgia S. Clarke, Jessica A. Grieger

**Affiliations:** ^1^ Faculty of Health and Medical Sciences Adelaide Medical School The University of Adelaide Adelaide Australia; ^2^ Robinson Research Institute The University of Adelaide Adelaide Australia; ^3^ Lifelong Health Theme South Australian Health and Medical Research Institute Adelaide Australia

**Keywords:** diet, gestational diabetes, maternal nutrition, metabolomics, pregnancy, systematic review

## Abstract

**Aim:**

To synthesise the evidence from clinical trials and observational studies using omics techniques to investigate the impact of diet and lifestyle factors on metabolite profile in pregnancy, and in the prevention and management of gestational diabetes mellitus (GDM).

**Materials and Methods:**

A systematic literature search was performed using PubMed, Ovid, CINAHL, and Web of Science databases in October 2023 and updated in September 2024. Inclusion criteria were randomised controlled trials (RCT) or non‐RCTs in pregnant women with or without GDM, that measured diet and lifestyle factors, and which applied post‐transcriptional omics approaches. Risk of bias was assessed using the ROBINS‐I for non‐RCTs and ROB‐2 tool for RCTs. The results of all studies are narratively synthesised.

**Results:**

Of 6293 studies identified, eight observational studies and three RCTs comprising 2639 pregnant women were included. Three studies reported on changes in diet‐related metabolic phenotypes during pregnancy; however, the impact of certain foods on the metabolome and risk for GDM was less clear. Compared with women without GDM, women with GDM had a worse deterioration in metabolites, including saturated fatty acids, branched chain amino acids and purine degradation metabolites. There is limited evidence that conventional dietary treatment for GDM may modify the metabolome in women with GDM.

**Conclusions:**

Metabolome profiles in pregnancy may be altered by certain dietary choices; however, it is inconclusive whether improved diet related metabolite profiles have a beneficial impact in the prevention or management of GDM. High quality studies with larger sample sizes are needed to better understand the role that maternal nutrition plays in modulating the maternal metabolome, not only for a healthy pregnancy but also for the prevention and management of GDM.

**Abbreviations**
GDM: Gestational diabetes mellitus
VLDL: Very‐low‐density‐lipoproteins

## 1 Introduction

In normal pregnancy, there are constant alterations in metabolism to support foetal growth and development, including increased insulin secretion and insulin resistance [1]. Gestational diabetes mellitus (GDM) is one of the most common disorders of pregnancy, affecting around 14% of pregnancies, globally, and occurs when there is an imbalance in insulin sensitivity and secretion [2]. Women diagnosed with GDM are not only at higher risk of pregnancy complications, such as pre‐eclampsia and deliver by caesarean section their infants are also more likely to be born large‐for‐gestational‐age [3, 4]. In the longer term, women who had GDM are at an eight‐fold increased risk for developing type 2 diabetes mellitus [5, 6] and a two‐fold increased risk of cardiovascular disease [7].

Diet and physical activity interventions reduce the risk for GDM by around 20%–30%, with greater reductions in risk depending on certain participant characteristics [8]. However, in population‐based pregnancy cohorts and also in studies investigating the prevention or management of GDM [9], food intake is not accurately defined or controlled, the dietary interventions and their dietary methods of assessment are heterogenous, and the optimal components that comprise an effective intervention are not known [10]. Thus, whilst objective biomarkers of dietary intake can provide an understanding of food intake and relationships to health outcomes, the identification of smaller metabolites, using omics techniques, may be used to uncover nutrition related metabolic pathways that may be associated with disease and that may not have been previously considered.

Omics refers to the study of all detectable biological molecules, including genes (genomics), transcripts (transcriptomics), proteins (proteomics), metabolites (metabolomics) and lipids (lipidomics) in body fluids or tissues, providing insights into the metabolic phenotype at the time of sample collection [11]. In normal pregnancy, omics studies have revealed increases in blood lipids, including triglycerides, very‐low‐density‐lipoproteins (VLDL), low‐density lipoproteins, and very large high‐density lipoprotein (HDL)‐particles, over gestation [12, 13], and increases in essential omega‐3 and omega‐6 fatty acids [12, 14]. Certain amino acid species have been shown to increase, decrease, or remain unchanged [12, 14, 15]. Such changes are in accordance with pregnancy being a hyperlipidemic and pro‐inflammatory state with alterations in metabolic and energy demands by the foetus. Metabolomic studies have also revealed that aromatic amino acids and branched chain amino acids, carbohydrates (e.g. glucose and hexose metabolism), and lipids (e.g. fatty acids, phospholipid and sphingolipid metabolism), are impaired in women with GDM [16, 17].

Nutritional intake is important in pregnancy and GDM, and the metabolome may be used as a proxy measurement to understand functional relationships between nutrients and health outcomes. However, there is no comprehensive synthesis of whether and how diet‐related metabolomic profile might be different over pregnancy and in women with GDM, and, whether the metabolomic profiles may be affected by other lifestyle behaviours such as physical activity. Improved understanding of the interaction between diet and lifestyle factors and metabolite profiles may generate improved and more personalised diet and lifestyle strategies for a healthy pregnancy and for the prevention and management of GDM. The research question is: How can diet or lifestyle related metabolites inform a healthy pregnancy and the prevention or management of GDM? This systematic review aimed to synthesise evidence from randomised controlled trials and observational studies that investigated diet or lifestyle factors and the association with metabolite profiles in pregnancy and in GDM; and whether dietary management for GDM influences dietary metabolite profiles.

## 2 Materials and Methods

The systematic review was registered with PROSPERO (International register of Systematic Reviews) and is accessible via the registration code: CRD42023467196. The study protocol is also provided as Electronic Supporting Information S1 (ESM 1). The review was conducted in line with the Cochrane and Preferred Reporting Items for Systematic reviews and Meta‐Analyses (PRISMA 2020) guidelines [18, 19]. An electronic PRISMA checklist for the manuscript and abstract is provided in EMS Tables 1 and 2.

**TABLE 1:** Characteristics of included studies.

First author, year, country, study design	Maternal age (years)	BMI (kg/m^2^)	Lifestyle data collection methods and time points	Biospecimen type, collection timepoints, type of omics	GDM diagnosis (criteria/%GDM cases)
Marchioro, 2019 Ireland RCT *n* = 51	Median (IQR) Intervention: 32.0 (4.2) years Secondary analysis Maternal blood Control: 33.37 (5.73) years Intervention: 32.30 (6.89) years	Median ± IQR Pre‐pregnancy: Intervention: 26.8 (5.1) kg/m^2^ Control: 26.8 (4.8) kg/m^2^ Secondary analysis Maternal blood: Control: 24.34 (4.40) kg/m^2^ Intervention: 26.36 (5.33) kg/m^2^	3‐day food diaries Collected at: 15 weeks' gestation 28 weeks' gestation 34 weeks' gestation	Plasma (Fasting) Collected at: Recruitment (median 13 weeks') 28 weeks' gestation Targeted metabolomics	2‐step GDM screening programme: −50 g OGTT (28 weeks') ‐ If 1 h plasma glucose > 8.3 mmol/L, then administered a 3 h 100 g OGTT using Carpenter‐Coustan criteria^a^ Number of GDM cases not reported
Mills, 2019 UK RCT *n* = 1158	**Control (n = 577):** 16.8% < 25 years; 24.4% 25–29 years; 32.4% 30–34 years; 26.3% ≥ 35 years **Intervention (n = 581):** 14.6% < 25 years; 28.4% 25–29 years; 29.9% 30–34 years; 27% ≥ 35 years	Pre‐pregnancy: **Control (n = 577):** 47.3% 30–34.9 kg/m^2^; 35.2% 35–39.9 kg/m^2^; 17.5% ≥ 40 kg/m^2^ **Intervention (n = 581):** 49.4% 30–34.9 kg/m^2^; 30.5% 35–39.9 kg/m^2^; 20.1% ≥ 40 kg/m^2^	Food frequency questionnaire International physical activity questionnaire Collected at: 15 + 0 and 18 + 6 weeks' gestation (Recruitment) 34 + 0 and 36 + 0 weeks' gestation	Serum (non‐fasting) Collected at: 15 + 0 and 18 + 6 weeks of gestation (recruitment) 34 + 0 and 36 + 0 weeks' gestation Targeted metabolomics	75 g OGTT (27–28 weeks') International association of the diabetes and pregnancy study groups (IADPSG) criteria^b^ GDM: 25.3% (*n* = 146) in control and 23.6% (*n* = 137) in control (11.3% data missing)
Sugino, 2024 United States RCT *n* = 34	Mean ± SD Control: 32.96 ± 3.08 years Intervention: 31.96 ± 4.77 years	Mean ± SD Maternal BMI at 30 weeks' gestation: Control: 31.47 ± 4.95 kg/m^2^ Intervention: 32.93 ± 5.69 kg/m^2^	Meals prepared by research centre and picked up by participants every 3 days	Plasma (fasting 10 h) Collected at: 30–31 weeks' gestation 36–37 weeks' gestation Targeted metabolomics	100 g OGTT (24–28 weeks) Carpenter‐Coustan criteria^a^ GDM cases: 100%
Chehab, 2023 United States Observational *n* = 261	7.6% < 25 years; 19.2% 25–29 years; 44.4% 30–34 years; 28.7% ≥ 35 years	Pre‐pregnancy: 1.9% < 18.5 kg/m^2^; 36.0% 18.5–24.9 kg/m^2^; 29.5% 25–29.9 kg/m^2^; 32.6% ≥ 30 kg/m^2^	Block food frequency questionnaire Pregnancy physical activity questionnaire Collected at: 10–13 weeks' gestation 24–28 weeks' gestation	Serum (fasting) Collected at: 10–13 weeks' gestation 24–28 weeks' gestation Untargeted metabolomics	2‐step GDM screening −50 g, 24 h glucose challenge ‐ If deemed with positive (glucose challenge test ≥ 7.8 mmol/L) then administered a 3 h, 100 g OGTT using Carpenter‐Coustan criteria^a^ GDM cases: 29.8% (*n* = 64)
Chen, 2010 United States Observational *n* = 227	Mean ± SE GDM: 25.63 ± 0.81 years Impaired GCT non‐GDM: 24.48 ± 0.65 years Normal GCT: 21.29 ± 0.54 years	Mean ± SE Pre‐pregnancy: GDM: 30.76 ± 0.93 kg/m^2^ Impaired GCT non‐GDM: 26.93 ± 0.71 kg/m^2^ Normal GCT: 25.40 ± 0.56 kg/m^2^	24‐h recall Collected at: 16.5 ± 0.16 weeks' gestation 20 weeks' gestation 28 weeks' gestation	Serum (fasting) Collected at: 16.5 ± 0.16 weeks' gestation 3rd trimester 30.76 ± 0.16 weeks' gestation Lipid profiling	2‐step GDM screening programme (28 weeks'): −50 g OGTT ‐ If 1 h plasma glucose > 7.8 mmol/L, then administered a 3 h 100 g OGTT using Carpenter‐Coustan criteria^a^ GDM cases: 27.5% (*n* = 40)
Chen, 2018 China Observational *n* = 93	Median (IQR) Non‐GDM: 29 (27; 33) years GDM: 32 (29; 34) years	Median (IQR) Pre‐pregnancy: Non‐GDM: 20.7 (19.0; 21.8) kg/m^2^ GDM: 21.6 (20.2; 24.6) kg/m^2^	Food frequency questionnaire Collected at: 24–28 weeks' gestation	Serum, urine and hair samples (unspecified if fasting/non‐fasting) Collected at: 24–28 weeks' gestation Untargeted metabolomics	75 g OGTT (24–28 weeks') IADPSG criteria^b^ GDM cases: 52.7% (*n* = 49)
Law, 2017 China Observational *n* = 61	Mean ± SD GDM: 29.0 ± 3.3 years Control: 26.9 ± 3.03 years	Mean ± SD **Pre‐pregnancy:** GDM: 22.0 ± 4.22 kg/m^2^ Control: 20.9 ± 2.15 kg/m^2^ **1st trimester** GDM: 22.9 ± 4.32 kg/m^2^ Control: 20.8 ± 2.41 kg/m^2^ **2nd trimester** GDM: 26.0 ± 4.17 kg/m^2^ Control: 23.5 ± 2.87 kg/m^2^ **3rd trimester** GDM: 27.6 ± 4.13 kg/m^2^ Control: 25.7 ± 3.44 kg/m^2^	Not reported GDM patients received standard dietary and/or lifestyle counselling upon diagnosis	Spot urine (after overnight fast) 3 visits, once each trimester Metabolomics (unspecified)	75 g OGTT (24–28 weeks') IADPSG criteria GDM cases: 44.26% (*n* = 27)
Mokkala, 2020 Finland Observational *n* = 352	Median (IQR) Non‐GDM: 30.2 (27.1; 33.4) years GDM: 31.0 (28.8; 34.8) yrs	Median (IQR) Non‐GDM: 28.4 (26.3; 31.2) kg/m^2^ GDM: 30.1 (27.2; 33.6) kg/m^2^	3‐day food diaries Collected at: 34–36 weeks' gestation	Serum (fasting > 10 h) Collected at: 34–36 weeks' gestation Targeted metabolomics	75 g OGTT (24–28 weeks') If one or more values were at or above the following threshold levels: Fasting plasma glucose ≥5.3 mmol/L; 1 h ≥ 10.0 mmol/L; 2 h ≥ 8.6 mmol/L GDM cases: 28.4% (*n* = 100)
Park, 2015 Korea Observational *n* = 89	Mean ± SD Non‐GDM: 33.3 ± 3.8 years GDM: 33.7 ± 4.1 yrs	Mean ± SD Pre‐pregnancy Non‐GDM: 20.5 ± 1.6 kg/m^2^ GDM: 24.0 ± 3.8 kg/m^2^	24‐h recall Collected at: 24–28 weeks' gestation	Plasma (unspecified fasting/non‐fasting) Collected at: 24–28 weeks' gestation Amino acid profiling	2‐step GDM screening programme (24–28 weeks' gestation) −50 g OGTT ‐ If 1 h plasma glucose > 7.8 mmol/L, then administered a 3 h 100 g OGTT using Carpenter‐Coustan criteria^a^ GDM cases: 71.9% (*n* = 64)
Pinto, 2016 Portugal Observational *n* = 98	Range: Controls 1 (*n* = 14): 24–39 years Non‐treated GDM: diet‐requiring (*n* = 12): 28–41 years Insulin‐requiring (*n* = 8): 38–41 years Controls 2 (*n* = 30): 20–39 years Diet‐treated GDM (*n* = 28): 18–41 years Insulin‐treated GDM (*n* = 8): 23–39 years	Range: Pre‐pregnancy: Controls 1 (*n* = 14): 16.9–32.0 kg/m^2^ Non‐treated GDM: diet‐requiring (*n* = 12): 17.6–35.8 kg/m^2^ Insulin‐requiring (*n* = 6): 20.0–30.9 kg/m^2^ Controls 2 (*n* = 30): 16.9–34.4 kg/m^2^ Diet‐treated GDM (*n* = 28): 18.1–40.4 kg/m^2^ Insulin‐treated GDM (*n* = 8): 28.1–44.7 kg/m^2^	Methods not reported Standard GDM dietary education: 1600–2000 kcal/d	Spot urine Collected at: First appointment after diagnosis and before treatment, 16–32 weeks' gestation Follow‐up appointments during course of treatment: −17–40 weeks' gestation (diet treated GDM, for 2–13 treatment weeks −17–38 weeks' gestation (insulin treated GDM) Metabolomics (unspecified)	75 g or 100 g OGTT (16–29 weeks' gestation) IADPSG criteria^b^ Non‐treated GDM cases: 58.8% (*n* = 20/34) Diet‐treated GDM cases: 42.4% (*n* = 28/30) Insulin‐treated GDM cases: 12.1% (*n* = 8/30)
van der Plight, 2023 Australia Observational *n* = 215	Mean ± SD 31.5 ± 3.9 years	Early pregnancy 25.0 ± 4.6 kg/m^2^	The dietary questionnaire for epidemiological studies version 2 Collected at: Early pregnancy: 15 ± 3 weeks' gestation Late pregnancy: 35 ± 2 weeks' gestation	Plasma (non‐fasting) Collected at: Early pregnancy: 15 ± 3 weeks' gestation Mid pregnancy: 27 ± 3 weeks' gestation Untargeted lipidomics	75 g OGTT (24–28 weeks') IADPSG criteria^b^ GDM cases: 9.7% (*n* = 21)

Abbreviations: BMI body mass index; GDM gestational diabetes mellitus; HP hypertension; IQR: inter‐quartile range; OGTT oral glucose tolerance test; RCT: randomised controlled trial.

^a^ Carpenter‐Coustan criteria: diagnosis of GDM with two or more glucose values over the cut points of 95, 180, 155, and 140 mg/dL at fasting, 1,2 and 3 h during a 100‐g diagnostic oral glucose tolerance test.

^b^ International Association of the Diabetes and Pregnancy study groups (IADPSG) criteria: diagnosis of GDM based on one of: fasting glucose ≥ 5.1 mmol/L, 1‐h level ≥ 10 mmol/L, or 2‐h level ≥ 8.5 mmol/L.

**TABLE 2:** Summary of findings from included studies.

Author	Diet	GDM	Metabolites	Outcome to pregnancy/GDM
Changes in diet related metabolites in normal pregnancy				
van der Plight, 2023	Australian dietary guideline index score	Not reported on	Total dietary guideline index score at early pregnancy was significantly associated with 7/113 plasma triglycerides at mid‐pregnancy, six of which were negative associations. Three triglycerides contained the 18:0 saturated fatty acid stearic acid. Total dietary guideline index score significantly positively associated with palmitoleic acid an unsaturated fat	Higher overall diet quality benefit to metabolite profile in later pregnancy.
Observational				
Mills, 2019	Intervention group: Intense behaviour change intervention, exchange carbohydrate‐rich foods from medium‐to‐high glycaemic index to lower glycaemic index, restrict saturated fat intake.	Not reported on	Intervention versus control: Reductions in the rate of increase of concentrations of all lipids, phospholipids and triglycerides in extremely large, very large, large and medium VLDL particles, except for total cholesterol and cholesterol esters in medium VLDL. Less of an impact on fatty acids.	Intense diet and lifestyle intervention benefit to women in later pregnancy.
RCT	Control group. Usual GDM management			
Marchioro, 2019	Intervention group: Low intensity, one education session about low glycaemic index diet from early pregnancy to later pregnancy.	Not reported on	Higher levels of non‐esterified fatty acids, 8 mid‐chain acylcarnitines, three lysophosphatidylcholines, 15 diacyl‐phosphatidylcholines and acyl‐alkyl‐phosphatidylcholines, and 12 sphingomyelins in the intervention group compared to standard care. Not significant after correcting for multiple comparisons.	Low intense diet and lifestyle intervention no benefit to women in later pregnancy.
RCT	Control group. Usual GDM management			
Dietary metabolites and GDM				
Chehab, 2023	Refined grains	Risk of GDM	17 metabolites associated with both refined grain intake and the risk of GDM (fatty acyls, glycerolipids, glycerophospholipids, sphingolipids, steroids, carboxylic acids, organooxygen compounds, phenylpropanoic acid clusters).	Inconsistent benefit from refined grain metabolites and risk for GDM.
Observational				
Chen, 2018	Snack‐based dietary pattern (fruits, yoghurt, sweet/savoury breads/buns, and low‐fat milk) related to a range of metabolites.	Risk for GDM	35/232 metabolites significantly different between GDM cases and non‐GDM controls. Not significant after adjustment for multiple comparisons.	No benefit from snack based dietary metabolites and risk for GDM.
Observational				
Chen, 2010	Weak positive correlations were observed between both serum and dietary intake of polyunsaturated fatty acids	Women with GDM, impaired glucose tolerance, or non‐GDM controls	In later pregnancy, weak positive correlations were observed between both serum and dietary intake of polyunsaturated fatty acids, irrespective of GDM status, despite the non‐GDM controls reporting a higher intake of dietary polyunsaturated fatty acids across pregnancy than women with impaired glucose tolerance.	Adverse lipid metabolite profile, regardless of GDM status.
Observational				
Park, 2015	No correlation between plasma amino acids and dietary protein intake.	GDM versus non GDM	Circulating aspartate, glycine, arginine, alanine, proline, lysine, and isoleucine levels at 24–28 weeks of pregnancy were significantly higher in GDM women than non‐GDM women which, although the women with GDM had higher dietary intakes of all 20 amino acids and protein than the non‐GDM group.	No benefit from diet related amino acid metabolite profile in women with GDM versus non GDM.
Observational				
Dietary intake may modify the metabolome in women with GDM				
Law, 2017	Standard dietary and/or lifestyle counselling upon GDM diagnosis.	Women with normal glucose tolerance and women with GDM.	Increased excretion of several metabolites of tryptophan metabolism and nucleosides of purine metabolism in women with GDM relative to normal glucose tolerance.	Adverse benefit to women with diet controlled GDM.
Observational				
Pinto, 2016	Diet treatment for women diagnosed with GDM (recommended 1600–2000 kcal diet).	GDM treatment	Stabilised creatinine, glucose and glutamine but not dimethylamine and succinate.	Metabolite benefits to women with diet controlled GDM.
Observational			Side effects to diet treatment were nucleotide metabolism, threonine metabolism [4‐deoxyerythronic acid (4‐DEA), 4‐deoxythreonic acid (4‐DTA)], methionine metabolism [dimethylglycine] (DMG)] and sugar metabolism (sucrose and mannose).	
Mokkala, 2020	Finnish Current care guidelines recommend carbohydrates with high fibre content; mono‐ and polyunsaturated fatty acids; increased fruits and vegetables.	In women with GDM	Women treated with medication had more pronounced lipid abnormalities (higher levels of VLDL particles, total lipids as well as higher cholesterol in VLDL particles and lower cholesterol in high density lipoprotein particles) compared to diet treated GDM women. Serum levels of leucine, isoleucine and glycoprotein acetylation also higher in women treated with medication than diet alone.	Balanced glucose metabolism in diet and medication treated GDM.
Observational				
Sugino, 2024	Intervention: Conventional high‐fat, 40% complex carbohydrate diet.	In women with GDM	Two metabolic clusters identified, but they were independent of diet treatment group: 1) relatively higher levels of fasting plasma triglycerides (higher total ceramides, total lysophosphatidylcholines, total triglycerides and total saturated and unsaturated triglycerides); 2) less of a decrease in fasting glucose.	No benefit from diet related carbohydrate‐metabolites to women with diet treated GDM.
RCT	Control: Low‐fat, 60% complex carbohydrate diet.			

### 2.1 Selection Criteria

The eligibility criteria were determined using the Participant‐Intervention/Exposure‐Comparison‐Outcome (PICO or PECO) framework. Participants (P): Participants included pregnant women with or without a diagnosis of GDM. Intervention (I) or Exposure (E): Intervention/exposure included intervention or observational studies that included diet or lifestyle variables (whole foods; nutrients; physical activity; sedentary activity; behavioural change; sleep and circadian rhythm). Studies included only post‐transcriptional omics approaches, such as proteomics, metabolomics and lipidomics analyses, as genetic variations, such as single nucleotide variants [20], may not always reflect differences in phenotypes [21]. Comparison (C): Comparisons included any comparison group for intervention trials (e.g. randomised control trials [RCT], cluster‐randomised trials and single‐arm interventions) or observational studies with or without a comparator group. Outcomes (O): Studies were included if they reported on GDM (as described below) or assessed diet‐related metabolites in pregnancy. Exclusion criteria were reviews, protocol papers and conference abstracts, if they specifically studied genes (e.g. transcriptome, RNA seq), if the whole population was recruited based on certain medications (e.g. insulin or metformin), and studies using supplements or meal replacements.

### 2.2 Core Outcomes

The primary outcomes of this review were biomarkers relevant to GDM (e.g. glycaemic control, glucose tolerance, insulin resistance), GDM incidence, or omics data (metabolomics, proteomics, lipidomics, or multi‐omics).

### 2.3 Search Strategy

A systematic search was conducted in four databases: PubMed, CINAHL (Ultimate), OVID (Embase) and Web of Science on 26th October 2023. Search terms were developed by JAG in consultation with an academic librarian within the University of Adelaide (ESM Table 3). Restrictions were applied to animal studies, conference abstracts/posters, and studies that were not published in English. Publications were included from inception until 26th October 2023 and the search was updated on 4th September 2024. Reviews were checked for any relevant references before excluding. All citations were exported to Endnote and duplicates were removed before being uploaded to Covidence. Title and abstract screening and full text screening were conducted in duplicate by the research team. Any discrepancy in screening was resolved by a third reviewer.

### 2.4 Quality Assessment

Studies included after full text screening underwent quality appraisal, which was conducted by two independent researchers, KL and GSC, and crosschecked by JAG. ROBINS‐I [22] was used to assess non‐RCT observational studies and ROB‐2 tool [23] was used to assess RCT studies. Studies that only used baseline data in an RCT were treated as an observational analysis and assessed using ROBINS‐I.

### 2.5 Data Extraction and Analysis

Data extraction was conducted in duplicate by KL, GSC, and JXGC. Conflicts were resolved between extractors. For included studies, data extracted included name of first author, publication year, study design, study duration/time point recruited, study setting, inclusion/exclusion criteria, demographics, dietary assessment, omics analysis methods, biospecimens collected, and GDM diagnosis and outcomes. The corresponding authors of the two studies were contacted for missing information to be included in this systematic review. A meta‐analysis of data was not feasible due to the heterogenous nature of the included studies; thus, we report a systematic narrative review of the characteristics and findings of the included studies.

## 3 RESULTS

**FIGURE 1:**
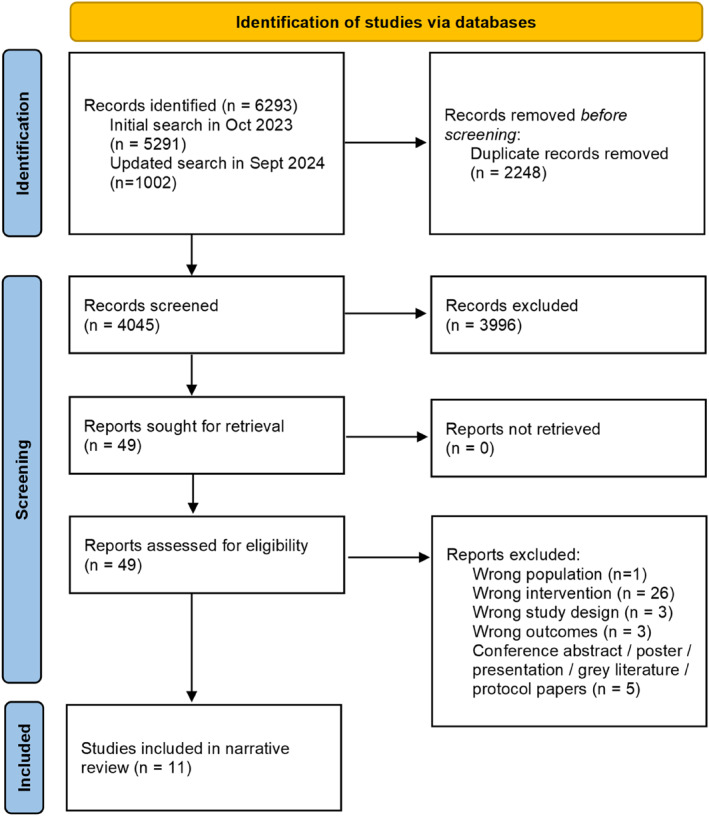
Prisma flow diagram.

Five thousand two hundred ninety one articles were identified through the initial systematic search and hand search. The updated search conducted in September 2024 identified another 1002 records. After duplicates were removed (*n* = 2248), 4045 articles were imported into Covidence, and 49 studies were assessed for eligibility (Figure 1). After excluding 36 articles in full‐text, one study was excluded as the corresponding author did not provide details regarding the outcome analysis [24], and one study was excluded as dietary intake was not analysed in relation to the metabolome [25]. A total of 11 studies (eight observational studies and three secondary analyses of RCTs) comprising 2639 pregnant women were synthesised.

All studies explored dietary factors and two additionally included a general ‘lifestyle’ or physical activity component [12, 26]. Three studies reported on changes in diet related metabolome over pregnancy [12, 13, 27]; four observational studies explored diet related metabolites and GDM [28, 29, 30, 31]; and four studies compared diet treatment versus control, in women with GDM, on the metabolome [26, 32, 33, 34]. Most studies adjusted for variables that may affect diet and the metabolic profile, including maternal age and pre‐pregnancy body mass index (BMI), two studies adjusted for BMI but not maternal age [27, 32], one study included physical activity as a confounder [28], and two studies did not adjust for any confounding variable [33, 34].

Study characteristics including participants mean age and BMI, lifestyle data collection methods, biospecimen collection, omics methods, and GDM diagnosis are reported in Table 1. Nine studies performed metabolomics analysis, including four targeted metabolomic analyses using serum or plasma samples [12, 27, 32, 34], two studies used untargeted metabolomic analyses with serum samples [28, 29], urine and hair [29], and two studies performed urine metabolome without specifying the type of metabolomic analysis [26, 33]. Two studies focused on lipids by conducting lipid profiling using serum [30] or untargeted lipidome using plasma samples [13] and one study conducted amino acid profiling with plasma samples [31]. Studies collected diet data using food frequency questionnaires [12, 13, 28, 29], 24 h recalls [30, 31] or food diaries [27, 32]. Two studies did not report on dietary intake data but reported to provide standard dietary education to women with GDM [26, 33]. One RCT provided all the meals during the study period and did not report on actual dietary intake [34] (Figure 1).

### 3.1 Risk of Bias Assessment

**FIGURE 2:**
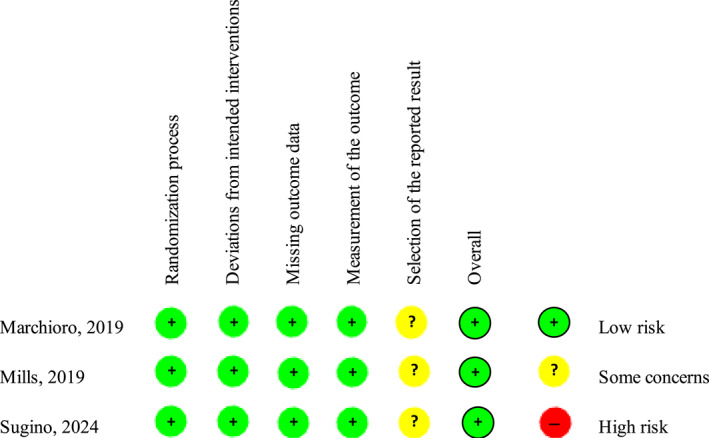
Risk of bias using the Cochrane risk of bias tool for randomised trials.

**FIGURE 3:**
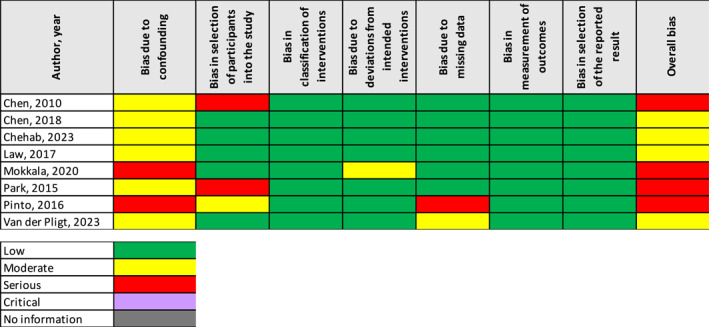
: Risk of bias for non‐randomised controlled trials using the ROBINS‐I tool.

Of the three RCTs that were included, all studies had a low risk of bias for each criterion, except for selection of the reported results, which all were identified as having some concerns. All three RCTs had an overall low risk of bias (Figure 2). For the eight non‐RCTs, all studies were identified to have moderate or be at serious risk of bias due to confounding factors (Figure 3). All studies had a low risk of bias in three criteria: bias in classification of interventions, bias in measurement of outcomes, and bias in selection of the reported results. Four studies were identified to have an overall moderate risk of bias and four studies had a serious risk of bias.

### 3.2 Changes in Diet Related Metabolites in Pregnancy

Table 2 provides a summary of findings from the included studies. One observational study and two RCTs examined diet and the metabolome over the course of pregnancy [12, 13, 27]. An observational study in 215 pregnant women living in Australia assessed diet quality during early pregnancy using the Australian Dietary Guideline Index [13]. Diet quality decreased over the course of pregnancy and most plasma lipids increased significantly from early to mid‐pregnancy. The total dietary guideline index score at early pregnancy was significantly associated with 7/113 plasma triglyceride species at mid‐pregnancy, six of which were negative associations, with adjustment for 0.2 false discovery rate (FDR). Three triglycerides contained the 18:0 saturated fatty acid, stearic acid. The total dietary guideline index score was significantly positively associated with palmitoleic acid, an unsaturated fatty acid [13].

An intense behaviour change intervention utilised lifestyle coaching to women with obesity that included increasing physical activity, exchanging carbohydrate‐rich foods with a medium‐to‐high glycaemic index for those with a lower glycaemic index, and restricting dietary intake of saturated fat, to reduce the incidence of GDM and large for gestational age infants [35]. The lifestyle intervention group demonstrated a dampened increase of all lipids, phospholipids and triglycerides in all large sized VLDL particles, saturated fatty acid proportions, lactate, pyruvate and alanine, and attenuated the reduced rate of linoleic, omega‐6 and polyunsaturated fatty acids from early to late pregnancy (16–36 weeks of gestation), compared to the rates of change in the control group [12]. The impact of the altered metabolome on GDM incidence was not assessed. Another RCT prescribed a low glycaemic index diet for a median 15 weeks of gestation to 34 weeks of gestation [27]. Compared to the high intensity intervention by Mills et al, higher levels of non‐esterified fatty acids, eight mid‐chain acylcarnitines, three lysophosphatidylcholines, 15 diacyl‐phosphatidylcholines and acyl‐alkyl‐phosphatidylcholines, and 12 sphingomyelins (with saturated or monounsaturated fatty acid chains) were found in the intervention group compared to standard care, however none remained significant after correcting for multiple comparisons [27].

### 3.3 Dietary Metabolites and GDM

Two observational studies in pregnant women explored diet related metabolome and risk for GDM with a further two studies examining diet related metabolome in women with GDM compared to women without GDM. In a case‐control metabolome‐wide association study nested within a population‐based longitudinal multiracial and ethnic pregnancy cohort, of the 821 annotated metabolites identified from metabolome‐wide association analysis, 42 were associated with refined grain intake after FDR adjustment, of which 17 of the metabolites were associated with risk for GDM, using chemical similarity enrichment analysis [28]. Overall, the glycerolipid cluster, specifically triacylglycerol 49:3, was associated with a doubled risk for GDM (OR: 2.15; 95%CI: 1.33, 3.48), whereas glycerophospholipids, specifically phosphatidylcholine 36:3 B (OR: 0.45; 95%CI: 0.28, 0.73) and sphingolipid clusters (driven by ceramide d34:0), that were also positively associated with refined grain intake, was associated with a lower risk for GDM (OR: 0.58; 95%CI: 0.38, 0.88) [28]. In a dietary pattern analysis among Chinese pregnant women, none of the metabolites (i.e. glucosyl‐3‐keto‐1,25R‐hexacosanediol, E−10‐Hydroxynortriptyline, PGF2alpha‐dihydroxypropanylamine, acorusnol, and tetradecanol), which were significantly associated with the snack‐based dietary pattern, characterised by frequent intake of fruits, yoghurt, sweet/savoury breads/buns, and low‐fat milk, were associated with GDM [29].

In later pregnancy, weak positive correlations were observed between serum and dietary intake of polyunsaturated fatty acids, irrespective of GDM status (all *r* < 0.30, for women with GDM, women with impaired glucose tolerance, and for non‐GDM controls), despite the non‐GDM controls reporting a higher intake of dietary polyunsaturated fatty acids across pregnancy than women with impaired glucose tolerance [30]. In a hospital based cohort study, circulating aspartate, glycine, arginine, alanine, proline, lysine, and isoleucine levels at 24–28 weeks of pregnancy were significantly higher in women with GDM than in women with normoglycemia; however, although the women with GDM had higher dietary intakes of all 20 amino acids and protein than the non‐GDM group, there was no correlation between plasma amino acids and dietary protein intake [31].

### 3.4 Dietary Intake May Modify the Metabolome in Women With GDM

In an exploratory analysis by Law et al., although the excretion of urinary metabolites related to tryptophan metabolism and purine nucleosides increased over pregnancy and were altered in women who developed GDM compared to women with normal glucose tolerance, treatment of GDM that included diet and lifestyle advice from a nutritionist to control blood glucose, did not attenuate these increases [26]. Comparatively, higher intake of vegetables and fruits, which were associated with higher urinary moupinamide (found in eggplant), 3‐(3‐methylbutylidene)‐1(3H)‐isobenzofuranone (found in green vegetables), were observed among women who received GDM dietary education versus non‐GDM controls [26].

In a sample of 98 women who were recruited between 16 and 40 weeks' gestation, compared to women without GDM, those who were newly diagnosed with GDM but not yet treated had lower 3‐hydroxyisovalerate, 2‐hydroxyisobutyrate, creatinine, hypoxanthine, dimethylamine, succinate, as well as higher glucose and glutamine in urine [33]. When compared with age‐matched women without GDM, diet treatment for women diagnosed with GDM (recommended 1600–2000 kcal diet) was shown to stabilise creatinine, glucose and glutamine but not dimethylamine and succinate. The latter two metabolites were stabilised with the addition of insulin to the diet treatment. Additionally, there were side effects of diet treatment on nucleotide metabolism (2‐Py, NMND), threonine metabolism (4‐deoxyerythronic acid [4‐DEA], 4‐deoxythreonic acid [4‐DTA]), methionine metabolism [dimethylglycine] (DMG)] and sugar metabolism (sucrose and mannose) [33].

Serum samples were obtained at ∼35 weeks's gestation from women who participated in a single‐centre mother‐infant dietary intervention utilising fish oil and probiotics [32]. Women with GDM and who were treated with medication had higher levels of VLDL particles (various sizes), total lipids as well as higher cholesterol in VLDL particles and lower cholesterol in HDL particles compared to women with GDM treated by diet (Finnish Current Care guidelines include carbohydrates with high fibre content; two‐thirds of mono‐ and polyunsaturated fatty acids; and increased intakes of fruits and vegetables) [32]. Serum levels of leucine, isoleucine and glycoprotein acetylation were also higher in women treated with medication than in those treated with diet alone. The aberrant metabolic profile in the women treated with medication was evident despite the fact that there were no differences in their serum glucose levels attributable to the treatment mode.

An RCT utilised a seven‐week GDM diet intervention comparing a conventional high‐fat, 40% complex carbohydrate intervention to a low‐fat, 60% complex carbohydrate diet [34]. Both diets had similar amounts of fibre and foods with low to moderate glycaemic index. Following both diets, two metabolic clusters were identified: one was associated with relatively higher levels of fasting plasma triglycerides (higher total ceramides, total lysophosphatidylcholines, total triglycerides and total saturated and unsaturated triglycerides), and the other was associated with less of a decrease in fasting glucose; however, this was independent of diet treatment group [34].

## 4 Discussion

There is consistent evidence to show that metabolite profiles change during pregnancy, and that metabolite profiles shift further in women with GDM. This review contributes new knowledge by being the first to comprehensively synthesise and examine whether metabolic signatures relate to diet and lifestyle factors in pregnant women and in women with GDM. Findings demonstrate that deterioration in metabolites over the course of pregnancy may be improved by an overall improved diet quality; however, the impact of diet‐related metabolite profile on risk for GDM is unclear. In women with GDM, standard GDM dietary management may alter the metabolite profile, but whether this is partly contributed by medications for GDM cannot be delineated. The impact of other lifestyle‐related factors on the metabolome cannot be ascertained from this review as no studies were identified. Inclusion of physical activity was incorporated in the RCT by Mills et al., but its impact on the metabolome remains unclear, and consistent with a recent review comprising few studies showing that physical activity may regulate metabolites in pregnancy [36].

Diet is one of the most influential and complex exogenous modulators of our metabolic phenotype due to the diverse impact of dietary patterns, specific foods or food compounds on our cellular processes [37]. Our review showed that higher adherence to the Australian dietary guideline index was strongly associated with triglyceride lipid species over pregnancy [13] and an intense lifestyle intervention that improved nutrient profile, that substituted foods from a higher to lower glycaemic index, and that increased physical activity, dampened lipids, phospholipids and triglyceride species [12]. However, a low intense intervention solely targeting lower glycaemic foods may not have the desired effect on the metabolite profile [27]. Thus, an overall healthier dietary approach, particularly intervening with intense nutritional support early in pregnancy, may modify lipid metabolites in pregnancy, a critical period when women are vulnerable to dyslipidaemia. Importantly, the altered lipid and triglyceride metabolites identified are also associated with increased insulin resistance and inflammation [38] and are associated with the development of atherosclerosis and cardiovascular disease [39]. Therefore, such dietary modification to alter these metabolites would be important for the prevention of GDM as triglycerides are strong predictors of GDM [40, 41]. From the limited studies in this review, however, inconsistent associations were found between refined grain metabolites, particularly those related to lipids and triglycerides, and risk for GDM [28] along with glycolipids related to a snack‐based dietary pattern [29], indicating the need for further, high quality, holistic, dietary pattern studies linking metabolic profile and risk for GDM.

Certain aromatic amino acids such as tryptophan are essential amino acids obtained from diet, whereas serotonin is synthesised from tryptophan and plays a role in feeding behaviour. Purines are involved in DNA/RNA synthesis, energy production and signalling transmission. Such classes of lipid and amino acid metabolites have been associated with improved insulin sensitivity in type 2 diabetes mellitus [42, 43] and altered lipid and amino acid metabolites following higher fibre and lower glycaemic foods in people with obesity [44, 45]. In this review, we found that in later pregnancy, women with GDM had higher urinary tryptophan levels, including its metabolites such as serotonin, and purine metabolites [26] and higher amino acid metabolites [31], compared with women without GDM. However, the altered metabolites were not related to dietary protein intake [31], nor were they attenuated with diet and lifestyle advice [26]. Urine samples taken between 2 and 13 weeks after diet treatment were also shown to stabilise creatinine biosynthesis, glycaemia, and glutamine metabolism, but not galactose metabolism, and may adversely affect pathways involved in nucleotide metabolism [33]. Furthermore, there was no difference in metabolite profile following 4–6 weeks of diet treatment in line with Finnish diabetes guidelines compared with medication treatment, even though both diet and medication balanced glucose metabolism, suggesting medication provided no benefit to correct the disturbed lipids and amino acid metabolic profile [32]. Therefore, despite some potential positive findings, the limited information and analysis regarding the foods and nutritional intake with the prescribed dietary interventions and the unclear timeframe for when dietary management commenced, renders it difficult to draw strong conclusions for the impact of diet‐related urinary or blood metabolites for GDM management. Given the natural shifts in metabolites across pregnancy, and the exacerbated metabolite profile in GDM, diet treatment prescribed to women with GDM may need to be intense and with high compliance, and additionally points to both early metabolic profiling [46] and progression to earlier diagnosis and treatment of GDM [47].

The impact of maternal diet on metabolome profiles extends beyond reducing metabolic burden of the mother but its potential impact on foetal health and development. Altered maternal metabolite profiles identified in this review have demonstrated similar types of amino acid and fatty acid groups that have been associated with higher birth weight and infant adiposity [48, 49, 50, 51, 52]. In women with GDM, elevated lipid profiles have also been associated with larger infant birthweight and abdominal circumference [52]. This is important given that a meta‐analysis of 16 studies reported a 14% higher likelihood of total cardiovascular disease with low birthweight ( < 2500 g) and an 8% higher likelihood with high birthweight ( > 4000 g) [53]. Data on the impact of lifestyle interventions on maternal metabolites and studies reporting on the association with infant outcomes were limited in the studies reviewed, but this was not a primary aim for this review. In the UPBEAT trial, Marchioro et al. showed that a low glycaemic diet had no effect on the cord metabolome, and from the same cohort, Poston et al. found that the dietary intervention had no impact on the incidence of large‐for‐gestational‐age infants [35]. Future studies are needed to determine the relevance of maternal diet, the maternal and cord blood metabolome, and its association with birth outcomes.

### 4.1 Strengths, Limitations and Future of Metabolomics Studies

Strengths of this systematic review include the utilisation of a clear criteria for selecting studies that were relevant to our research question, for example, selecting post‐transcriptional omics techniques since they better reflect diet‐induced changes. The protocol was pre‐registered thereby minimising reporting bias, and we utilised transparent and reproducible methods for assessing the quality or risk of bias of the included studies.

There are several limitations of the included literature which should be considered. The studies were heterogeneous, including differences in biospecimen type (urine vs. plasma), collection time point (fasting, post‐prandial, or unspecified) and time point in gestation that samples were collected. Different dietary collection methods were utilised, for example, 3‐day food diary, food frequency questionnaire, or 24 h recall, which could contribute to exposure and self‐report biases. The criteria used for diagnosing GDM varied across the included studies and one study did not specify when in pregnancy the diagnosis was made. Limitations of the literature reviewed also include the overall concerning or serious risk of bias of included observational studies, mainly because of the lack of adjustment for confounding factors.

Future studies should include larger populations which are more ethnically diverse and include a more rigorous methodological design relating to dietary and biospecimen collection. Furthermore, with the advance of bioinformatics and machine learning and multi‐omics, the integration of different omics platforms has the potential to facilitate clinical judgement on diseases diagnosis, prognosis and treatment strategy [54]. As single omics approaches may only reflect part of the complex pathophysiological process [55], multi‐omics may generate a more holistic picture of the metabolic signature of diet‐health interactions for a healthy pregnancy but also for the prevention and management of GDM. Studies examining several biological fluids concurrently may provide a more complete overview of how diet and lifestyle impact metabolic phenotype in women with or without GDM. Additionally, inter‐generational omics studies are warranted to advance the understanding of diet/lifestyle impact on the intrauterine environment for foetal growth.

## 5 Conclusion

Shifts in metabolic profiles that naturally occur in pregnancy may be altered by dietary changes such as improving overall diet quality; however, the impact of diet on modulating metabolites to reduce the risk for GDM is unclear. There is limited evidence that dietary treatment may modify the metabolome in women with GDM. High quality studies with larger sample sizes are needed to better understand the role that maternal nutrition plays in modulating the maternal metabolome, not only for a healthy pregnancy but also for the prevention and management of GDM, and subsequent offspring health outcomes.

## Author Contributions

JAG conceived the conception and design of the review with input from KL and GSC. KL, GSC, and JAG undertook title and abstract screening and all authors contributed to full text screening and data extraction. KL and GSC reviewed and synthesised the extracted data and they both interpreted the data and wrote the first draft of the manuscript. JAG provided critical intellectual input into the manuscript with revisions. All authors read and approved the final version to be published.

## Conflicts of Interest

The authors declare no conflicts of interest.

### Peer Review

The peer review history for this article is available at https://www.webofscience.com/api/gateway/wos/peer-review/10.1002/dmrr.70026.

## Supporting information

Supporting Information S1

## Data Availability

No new data were generated or analysed in support of this work. All data are available via published manuscripts cited in this article.
