# Supplementary material for: The Use of Omics in Untangling the Effect of Lifestyle Factors in Pregnancy and Gestational Diabetes: A Systematic Review
Source: Diabetes Metab Res Rev. 2025 Jan 12;41(1):e70026. doi: 10.1002/dmrr.70026 (PMC11725626; doi:10.1002/dmrr.70026)
Supplement: Supplementary file 1 — Supporting Information S1 [file DMRR-41-e70026-s001.docx]

# **Electronic Supplementary Material: The use of omics in untangling the effect of lifestyle factors in pregnancy and gestational diabetes: a systematic review**

**ESM 1: PROSPERO systematic review protocol**

**Title: Omics application in untangling the effect of lifestyle interventions for gestational diabetes: a systematic review**

Review methods were amended after registration. Please see the revision notes and previous versions for detail.

**Research question:**

How can the omics profile inform prevention (preconception or early pregnancy) and management of GDM?

**Aim of the review:**

The primary aim of this systematic review is to explore the differences in proteins and metabolites (e.g. carbohydrate, amino acid, lipid species) associated with lifestyle changes among women with or without GDM.

**Searches:**

PubMed: Species=human; Article types=journal article; Languages=English

Web of Science: Languages=English; Document type=Article

Ovid Embase: Languages=English

CINAHL: Academic journals; Languages=English

**Key terms:** gestational diabetes, metabolomics

**Types of study to be included [1 change]**

Experimental (e.g. randomised control trials, cluster-randomised trials, single-arm interventions, and protocol papers of experimental studies) and observational studies (e.g. cross-sectional studies, prospective/retrospective cohort studies, case-control studies) that collected lifestyle data or measured lifestyle related exposures with the outcome of GDM, or in women who have GDM.

Omics studies will be excluded if they are specifically studying genes e.g. transcriptome, RNA seq etc. Conference abstracts, thesis, government report, reviews, commentary will be excluded.

**Condition or domain being studied**

Gestational diabetes

**Participants/population [1 change]**

Women of child-bearing age, currently pregnant or pregnancy planning (interconception period).

Studies will be excluded if the whole population is recruited based on certain medication (e.g. insulin or metformin).

**Intervention(s), exposure(s) [1 change]**

Lifestyle: dietary (whole food, nutrients), physical activity, sedentary activity, behavioural change, sleep, circadian rhythm (e.g. change in hormones). Omics: proteome, lipidome, metabolome and multi-omics.

Exclude: medical treatment; use of supplement or medications; studies that used meal replacements (e.g. shakes) rather than whole foods.

**Comparators/controls [1 change]**

Active controls (more than one active counselling session, or on a different treatments/supplements), then only the active intervention group that fits in with our inclusion criteria will be included in the data extraction.

**Context**

Dietary intakes, sleep data, physical activity data

**Main outcome(s) [1 change]**

Biomarkers relevant to GDM (e.g. glycaemic control, glucose tolerance, insulin resistance), GDM incidence, omics data (metabolomics, proteomics, lipidomics, or mult-omics).

Measures of effect: GDM incidence or GDM-related risk factors (e.g. glucose tolerance, insulin sensitivity/resistance)

**Additional outcome(s):**

None

**Data extraction (selection and coding) [1 change]**

All citations (titles and abstracts) identified will firstly be imported into an EndNote file where duplicates will be removed, and then exported into Covidence. All authors will contribute to screening (in duplicate), using a screening checklist to confirm whether the articles meet review inclusion and exclusion criteria. Any discrepancies with the screening will be resolved through discussion by JG. A 10% sample of all excluded articles will be cross-checked by JG. A copy of full text of papers will be obtained for each of the included studies. The screening checklist will be reapplied in assessing the content of the paper. Studies not meeting the review exclusion/inclusion criteria will be excluded. Data extraction of the included articles will be entered into an Excel spreadsheet that includes, for example:

- author, title, year
- study design, sample size
- maternal population characteristics: maternal age, week’s gestation, type of nutritional assessment
- omics application
- GDM rates/prevalence/risk

**Risk of bias (quality) assessment**

Quality of the included studies will be critically appraised using the Revised Cochrane Risk of Bias Tool for Randomized Trials (RoB 2.0) and ROBINS-I tool for non-RCTs.

**Strategy for data synthesis**

Data extraction tables will be used to describe and synthesize the findings of the included studies. A table of quality and characteristics of included studies will be used to provide an overview of the type of studies identified. Meta-analysis will not be conducted due to likelihood of small number of included studies.

**Analysis of subgroups or subsets [1 change]**

Subgroup analyses may be performed for patient characteristics including BMI, PCOS, or any other relevant conditions.

**Contact details for further information**

Jessica Grieger

jessica.grieger@adelaide.edu.au

**Organisational affiliation of the review**

University of Adelaide

**Review team members and their organisational affiliations**

Dr Jessica Grieger, The University of Adelaide
Dr Kai Liu, The University of Adelaide

**Type and method of review [1 change]**

Systematic review

**Anticipated or actual start date**

30 September, 2023

**Anticipated completion date**

31 December, 2024

**Funding sources/sponsors**

None

**Conflicts of interest**

None

**Language**

English

**Country**

Australia

**Date of registration in PROSPERO**

08 October 2023

**Date of first submission**

28 September 2023

**Revision note**

Added in Key terms, and Effect measures, as these weren't originally specified.2. Added a study exclusion: Omics studies will be excluded if they are specifically studying genes e.g. transcriptome, RNA seq etc.3. Added a participant exclusion: Studies will be excluded if the whole population is recruited based on certain medication (e.g. insulin or metformin).

**ESM: Table 1: PRISMA checklist for systematic review abstract**

| **Section and Topic** | **Item #** | **Checklist item** | **Reported (Yes/No)** |
| --- | --- | --- | --- |
| **TITLE** | | |  |
| Title | 1 | Identify the report as a systematic review. | Yes |
| **BACKGROUND** | | |  |
| Objectives | 2 | Provide an explicit statement of the main objective(s) or question(s) the review addresses. | Yes |
| **METHODS** | | |  |
| Eligibility criteria | 3 | Specify the inclusion and exclusion criteria for the review. | Yes |
| Information sources | 4 | Specify the information sources (e.g. databases, registers) used to identify studies and the date when each was last searched. | Yes |
| Risk of bias | 5 | Specify the methods used to assess risk of bias in the included studies. | Yes |
| Synthesis of results | 6 | Specify the methods used to present and synthesise results. | Yes |
| **RESULTS** | | |  |
| Included studies | 7 | Give the total number of included studies and participants and summarise relevant characteristics of studies. | Yes |
| Synthesis of results | 8 | Present results for main outcomes, preferably indicating the number of included studies and participants for each. If meta-analysis was done, report the summary estimate and confidence/credible interval. If comparing groups, indicate the direction of the effect (i.e. which group is favoured). | Yes |
| **DISCUSSION** | | |  |
| Limitations of evidence | 9 | Provide a brief summary of the limitations of the evidence included in the review (e.g. study risk of bias, inconsistency and imprecision). | Yes |
| Interpretation | 10 | Provide a general interpretation of the results and important implications. | Yes |
| **OTHER** | | |  |
| Funding | 11 | Specify the primary source of funding for the review. | No |
| Registration | 12 | Provide the register name and registration number. | Yes |

*From:*  Page MJ, McKenzie JE, Bossuyt PM, Boutron I, Hoffmann TC, Mulrow CD, et al. The PRISMA 2020 statement: an updated guideline for reporting systematic reviews. BMJ 2021;372:n71. doi: 10.1136/bmj.n71

**ESM: Table 2: PRISMA checklist for systematic review manuscript**

| **Section and Topic** | **Item #** | **Checklist item** | **Location where item is reported** |
| --- | --- | --- | --- |
| **TITLE** | | |  |
| Title | 1 | Identify the report as a systematic review. | Pg.1 |
| **ABSTRACT** | | |  |
| Abstract | 2 | See the PRISMA 2020 for Abstracts checklist. | ESM: Table 1 |
| **INTRODUCTION** | | |  |
| Rationale | 3 | Describe the rationale for the review in the context of existing knowledge. | Pg. 4 |
| Objectives | 4 | Provide an explicit statement of the objective(s) or question(s) the review addresses. | Pg. 5 |
| **METHODS** | | |  |
| Eligibility criteria | 5 | Specify the inclusion and exclusion criteria for the review and how studies were grouped for the syntheses. | Pg. 6 |
| Information sources | 6 | Specify all databases, registers, websites, organisations, reference lists and other sources searched or consulted to identify studies. Specify the date when each source was last searched or consulted. | Pg. 7 |
| Search strategy | 7 | Present the full search strategies for all databases, registers and websites, including any filters and limits used. | Pg.7 and ESM Table 3 |
| Selection process | 8 | Specify the methods used to decide whether a study met the inclusion criteria of the review, including how many reviewers screened each record and each report retrieved, whether they worked independently, and if applicable, details of automation tools used in the process. | Pg. 7 |
| Data collection process | 9 | Specify the methods used to collect data from reports, including how many reviewers collected data from each report, whether they worked independently, any processes for obtaining or confirming data from study investigators, and if applicable, details of automation tools used in the process. | Pg. 7 |
| Data items | 10a | List and define all outcomes for which data were sought. Specify whether all results that were compatible with each outcome domain in each study were sought (e.g. for all measures, time points, analyses), and if not, the methods used to decide which results to collect. | Pg. 7 |
|  | 10b | List and define all other variables for which data were sought (e.g. participant and intervention characteristics, funding sources). Describe any assumptions made about any missing or unclear information. | Pg. 7 |
| Study risk of bias assessment | 11 | Specify the methods used to assess risk of bias in the included studies, including details of the tool(s) used, how many reviewers assessed each study and whether they worked independently, and if applicable, details of automation tools used in the process. | Pg. 7 |
| Effect measures | 12 | Specify for each outcome the effect measure(s) (e.g. risk ratio, mean difference) used in the synthesis or presentation of results. | NA |
| Synthesis methods | 13a | Describe the processes used to decide which studies were eligible for each synthesis (e.g. tabulating the study intervention characteristics and comparing against the planned groups for each synthesis (item #5)). | Pg. 7 |
|  | 13b | Describe any methods required to prepare the data for presentation or synthesis, such as handling of missing summary statistics, or data conversions. | NA |
|  | 13c | Describe any methods used to tabulate or visually display results of individual studies and syntheses. | Pg. 7 |
|  | 13d | Describe any methods used to synthesize results and provide a rationale for the choice(s). If meta-analysis was performed, describe the model(s), method(s) to identify the presence and extent of statistical heterogeneity, and software package(s) used. | Pg. 7 |
|  | 13e | Describe any methods used to explore possible causes of heterogeneity among study results (e.g. subgroup analysis, meta-regression). | NA |
|  | 13f | Describe any sensitivity analyses conducted to assess robustness of the synthesized results. | NA |
| Reporting bias assessment | 14 | Describe any methods used to assess risk of bias due to missing results in a synthesis (arising from reporting biases). | Pg. 7 |
| Certainty assessment | 15 | Describe any methods used to assess certainty (or confidence) in the body of evidence for an outcome. | NA |
| **RESULTS** | | |  |
| Study selection | 16a | Describe the results of the search and selection process, from the number of records identified in the search to the number of studies included in the review, ideally using a flow diagram. | Pg.8 and Figure 1 |
|  | 16b | Cite studies that might appear to meet the inclusion criteria, but which were excluded, and explain why they were excluded. | Pg. 8 |
| Study characteristics | 17 | Cite each included study and present its characteristics. | Pg. 8 and Table 1 |
| Risk of bias in studies | 18 | Present assessments of risk of bias for each included study. | Pg.9 and Figure 2 & 3 |
| Results of individual studies | 19 | For all outcomes, present, for each study: (a) summary statistics for each group (where appropriate) and (b) an effect estimate and its precision (e.g. confidence/credible interval), ideally using structured tables or plots. | NA |
| Results of syntheses | 20a | For each synthesis, briefly summarise the characteristics and risk of bias among contributing studies. | Pg.9 and Figure 2 & 3 |
|  | 20b | Present results of all statistical syntheses conducted. If meta-analysis was done, present for each the summary estimate and its precision (e.g. confidence/credible interval) and measures of statistical heterogeneity. If comparing groups, describe the direction of the effect. | NA |
|  | 20c | Present results of all investigations of possible causes of heterogeneity among study results. | NA |
|  | 20d | Present results of all sensitivity analyses conducted to assess the robustness of the synthesized results. | NA |
| Reporting biases | 21 | Present assessments of risk of bias due to missing results (arising from reporting biases) for each synthesis assessed. | Figure 2 & 3 |
| Certainty of evidence | 22 | Present assessments of certainty (or confidence) in the body of evidence for each outcome assessed. | NA |
| **DISCUSSION** | | |  |
| Discussion | 23a | Provide a general interpretation of the results in the context of other evidence. | Pg. 12-13 |
|  | 23b | Discuss any limitations of the evidence included in the review. | Pg. 14-15 |
|  | 23c | Discuss any limitations of the review processes used. | Pg. 14-15 |
|  | 23d | Discuss implications of the results for practice, policy, and future research. | Pg. 15 |
| **OTHER INFORMATION** | | |  |
| Registration and protocol | 24a | Provide registration information for the review, including register name and registration number, or state that the review was not registered. | Pg. 6 |
|  | 24b | Indicate where the review protocol can be accessed, or state that a protocol was not prepared. | Pg. 6 |
|  | 24c | Describe and explain any amendments to information provided at registration or in the protocol. | EMS 1 |
| Support | 25 | Describe sources of financial or non-financial support for the review, and the role of the funders or sponsors in the review. | Pg.16 |
| Competing interests | 26 | Declare any competing interests of review authors. | Pg. 1 |
| Availability of data, code and other materials | 27 | Report which of the following are publicly available and where they can be found: template data collection forms; data extracted from included studies; data used for all analyses; analytic code; any other materials used in the review. | Pg. 16 |

*From:*  Page MJ, McKenzie JE, Bossuyt PM, Boutron I, Hoffmann TC, Mulrow CD, et al. The PRISMA 2020 statement: an updated guideline for reporting systematic reviews. BMJ 2021;372:n71. doi: 10.1136/bmj.n71

**ESM: Table 3: Search strategies used for the systematic review.**

| Database | Search terms |
| --- | --- |
| Pubmed | Diabetes, Gestational” [mh] OR “Diabetes, Gestational” [tiab] OR “Gestational Diabetes Mellitus” [tiab] OR “Gestational Diabetes” [tiab] OR “GDM” [tiab] OR “Gestational hyperglycemia” [tiab] OR “Gestational hyperglycaemia” [tiab] OR “Hyperglycemia in pregnancy” [tiab] OR “Hyperglycaemia in pregnancy” [tiab] OR “Diabetes in pregnancy” [tiab]OR “Diabetes mellitus in pregnancy” [tiab]  OR “Insulin Resistan*” [tiab] OR “Glucose Intoleran*” [tiab] OR “Insulin Sensitiv*” [tiab] OR “Glucose toleran*” [tiab]OR "diabetes pregnancy"[tiab:~3] OR "diabetes pregnant"[tiab:~3] OR "diabetic pregnancy"[tiab:~3] OR "diabetic pregnancies"[tiab:~3] OR "diabetic pregnant"[tiab:~3] OR "Diabetes pregnancies"[tiab:~3] OR "diabetes pregnant"[tiab:~3] OR "hyperglycaemia pregnancy"[tiab:~3] OR "hyperglycaemic pregnancy"[tiab:~3] OR "hyperglycaemic pregnancies"[tiab:~3] OR "hyperglycemic pregnancies"[tiab:~3] OR "hyperglycaemic pregnant"[tiab:~3] OR "hyperglycemic pregnant"[tiab:~3] AND “Metabolomics” [mh] OR "multiomics"[mh] OR “metabolomics” [Text Word] OR "metabolomics" [MeSH Terms] OR “metabolo*” [All Fields] OR “metabonom*” [All Fields] OR “metabolite network*” [All Fields] OR “metabolite profile*” [All Fields] OR “lipidom*” [All Fields] OR "proteome"[MeSH Terms] OR "proteomics" [MeSH Terms] OR “proteom*” [All Fields] |
| OVID (Embase) | Exp Pregnancy diabetes mellitus OR Pregnancy diabetes mellitus.ti,ab OR Gestational Diabetes Mellitus.ti,ab OR Gestational Diabetes.ti,ab  OR GDM.ti,ab OR Gestational hyperglycemia.ti,ab OR Gestational hyperglycaemia.ti,ab OR Hyperglycemia in pregnancy.ti,ab OR Hyperglycaemia in pregnancy.ti,ab OR Diabetes in pregnancy.ti,ab OR Diabetes mellitus in pregnancy.ti,ab OR Insulin Resistan*.ti,ab OR Glucose Intoleran*.ti,ab OR Insulin Sensitiv*.ti,ab OR Glucose Toleran*.ti,ab OR ((Diabet* OR hyperglyc?emi*) adj4 pregnan*).ti,ab AND Omics.sh OR metabolomics.sh OR nutrimetabolomics.sh OR metabonomics.sh OR multiomics.sh OR nutriomics.sh OR foodomics.sh OR metabolomics.ti,ab OR metabolo*.ti,ab OR metabonom* .ti,ab OR metabolite network.ti,ab OR metabolite profile*.ti,ab OR lipidom*.ti,ab OR proteome.ti,ab OR proteomics.ti,ab OR proteom*.ti,ab |
| CINAHL | MH “Diabetes Mellitus, Gestational” OR TI “Diabetes Mellitus, Gestational” OR AB “Diabetes Mellitus, Gestational” OR TI “Gestational Diabetes Mellitus” OR AB “Gestational Diabetes Mellitus” OR TI “Gestational Diabetes” OR AB “Gestational Diabetes” OR TI “GDM” OR AB “GDM” OR TI “Gestational hyperglycemia” OR AB “Gestational hyperglycemia” OR TI “Gestational hyperglycaemia” OR AB “Gestational hyperglycaemia” OR TI “Hyperglycemia in pregnancy” OR AB “Hyperglycemia in pregnancy” OR TI “Hyperglycaemia in pregnancy” OR AB “Hyperglycaemia in pregnancy” OR TI “Diabetes in pregnancy” OR AB “Diabetes in pregnancy” OR TI “Diabetes mellitus in pregnancy” OR AB “Diabetes mellitus in pregnancy” OR TI “Insulin Resistan*” OR AB “Insulin Resistan*” OR TI “Insulin Sensitiv*” OR AB “Insulin Sensitiv*” OR TI “Glucose Intoleran*” OR AB “Glucose Intoleran*” OR TI “Glucose toleran*” OR AB “Glucose toleran*” OR TI ((Diabet* OR hyperglyc#emi*) N4 pregnan*) OR AB ((Diabet* OR hyperglyc#emi*) N4 pregnan*) **AND** MH metabolomics OR MH multiomics OR TI “metabolomics” OR AB “metabolomics" OR TI “metabolo*” OR AB “metabolo*” OR TI “metabonom*” OR AB “metabonom*” OR TI “metabolite network*” OR AB “metabolite network*” OR TI “metabolite profile*” OR AB “metabolite profile*” OR TI “lipidom*” OR AB “lipidom*” OR TI "proteome" OR AB "proteome" OR TI "proteomics" OR AB "proteomics" OR TI “proteom*” OR AB “proteom*” |
| Web of Science | *Under titles:*  “Gestational Diabetes Mellitus” OR “Gestational Diabetes” OR GDM  OR “Gestational hyperglycemia” OR “Gestational hyperglycaemia” OR “Hyperglycemia in pregnancy” OR “Hyperglycaemia in pregnancy” OR “Diabetes in pregnancy” OR “Diabetes mellitus in pregnancy” OR “Insulin Resistan*” OR “Insulin Sensitiv*” OR “Glucose Intoleran*” OR “Glucose toleran*” AND metabolomics OR metabolo*OR metabonom* OR “metabolite network*” OR “metabolite profile*”OR lipidom* OR proteome OR proteomics OR proteome* OR multiomics  *Under abstracts:*  “Gestational Diabetes Mellitus” OR “Gestational Diabetes” OR GDM OR “Gestational hyperglycemia” OR “Gestational hyperglycaemia” OR “Hyperglycemia in pregnancy” OR “Hyperglycaemia in pregnancy” OR “Diabetes in pregnancy” OR “Diabetes mellitus in pregnancy” OR “Insulin Resistan*” OR “Insulin Sensitiv*” OR “Glucose Intoleran*” OR “Glucose toleran*” AND metabolomics OR metabolo* OR “metabonom*” OR “metabolite network*” OR “metabolite profile*” OR lipidom* OR proteome OR proteomics OR proteom* OR multiomics |
